# CBCT-based virtual surgical planning for predicting risk factors and preventive strategies of buccal plate perforation in maxillary premolar implant placement

**DOI:** 10.1186/s13005-026-00611-3

**Published:** 2026-03-18

**Authors:** Weihua Zhu, Shengjun Yang, Peng Lin, Zaiou Zhu, Xiaomeng Song

**Affiliations:** 1 Department of Oral and Maxillofacial Surgery, The Affiliated Stomatological Hospital of Nanjing Medical University, Nanjing, Jiangsu 210029 People’s Republic of China; 2 Department of Oral and Maxillofacial Surgery, Stomatological Hospital Affiliated Suzhou Vocational Health College, Suzhou, Jiangsu 215002 People’s Republic of China; 3 State Key Laboratory Cultivation Base of Research, Prevention and Treatment for Oral Diseases, Nanjing Medical University, Nanjing, Jiangsu 210029 People’s Republic of China; 4 Jiangsu Province Engineering Research Center of Stomatological Translational Medicine, Nanjing Medical University, Nanjing, Jiangsu 210029 People’s Republic of China; 5 Department of Oral and Maxillofacial Surgery, The Affiliated Stomatological Hospital of Xuzhou Medical University, 130 West Huaihai Road, Xuzhou, Jiangsu 221002 People’s Republic of China

**Keywords:** Maxillary premolars, Virtual surgical planning, Buccal plate perforation, Cone beam computed tomography

## Abstract

**Background:**

Buccal plate perforation (BPP) is a common intraoperative complication during implant placement in the maxillary premolar region, compromising implant stability and long-term success. This study aimed to identify CBCT-based risk factors for BPP and develop a predictive model to optimize implant planning.

**Materials and methods:**

A retrospective analysis of cone beam computed tomography (CBCT) scans from 252 patients with missing maxillary premolars was performed. Virtual implant simulation evaluated the effects of alveolar bone type, alveolar bone inclination angle (ABIA), tooth position, and implant-to-bone angulation (IBA) on BPP incidence. Significant variables identified by Chi-squared and *t*-tests were further analyzed using multivariate logistic regression and receiver operating characteristic (ROC) analysis to determine predictive thresholds.

**Results:**

Sex, tooth position, alveolar bone type, ABIA, and IBA were significantly associated with BPP (*P* < 0.05). Female patients, first premolar sites, and buccally projected-concave bone types showed higher risk. BPP risk increased when ABIA ≥ 24.94° or IBA ≥ 20.71°, whereas a 1 mm palatal shift from the alveolar ridge center reduced its incidence from 34.69% to 7.1% with minimal palatal perforation (2.6%).

**Conclusions:**

CBCT-based virtual planning enables individualized risk assessment and optimization of implant trajectory. This study identifies key quantitative thresholds for ABIA and IBA, which, when combined with minor adjustments in implant positioning, provide a robust strategy for improving surgical outcomes. Although these findings are derived from virtual simulations, clinical validation is required to confirm their applicability in real-world clinical settings.

**Supplementary Information:**

The online version contains supplementary material available at 10.1186/s13005-026-00611-3.

## Introduction

Implant-supported prosthetic rehabilitation has become a widely accepted solution for managing partial edentulism, owing to its excellent long-term survival rate, ability to restore masticatory function, and improving patients’ quality-of-life [1]. However, implant therapy is not without complications. A comprehensive preoperative risk assessment is essential to ensure that the benefits of restoration outweigh the potential risks. This assessment should extend beyond bone volume to include patient-specific factors (e.g., age and sex) as well as key anatomical variables (e.g., alveolar bone morphology and inclination). Complications such as implant misalignment or improper angulation often arise from inadequate planning and can result in both esthetic and biomechanical compromise [2–4].

The maxillary premolar region presents significant anatomical challenges due to its complex morphology, relatively thin cortical bone, and frequent alveolar ridge inclination, making implant placement technically demanding [5]. Buccal plate perforation (BPP), a frequent intraoperative complications in this region [6, 7], undermines primary implant stability and may result in localized bone resorption, soft tissue dehiscence, and infection, thereby compromising long-term implant success and complicating esthetic and prosthetic outcomes [8].

Implant-related perforations in the maxilla have been reported in up to 43.5% of cases [9], with the premolar region particularly susceptible due to anatomical factors such as thin buccal bone and various alveolar contours [5]. Additionally, Alqutaibi et al. [6] conducted a virtual implant simulation study and reported a 47.6% incidence of BPP in the maxillary premolar region. Accordingly, anatomy-based individualized planning is essential for reducing the risk of BPP and improving implant-related outcomes [10].

Although previous studies have explored implant angulation, bone volume, and surgical guides in preventing perforation, most have focused on anterior or molar regions [11, 12], with systematic investigations of the maxillary premolar region remaining scarce. While CBCT-based virtual planning enables quantitative preoperative assessment of anatomical variables, current evidence supporting predictive modeling and intervention strategies is still insufficient. Moreover, most existing virtual planning studies have relied on CBCT scans from dentate patients, without accounting for post-extraction alveolar remodeling [13, 14]. However, significant anatomical changes occurring during the healing phase after tooth extraction, potentially resulting in discrepancies between virtual planning and actual clinical conditions [15].

Therefore, the present study utilized CBCT data from patients at least three months post-maxillary premolar extraction. Using virtual implant simulation, we aimed to identify risk factors for BPP from a broad range of patient-specific and radiographic parameters, to determine independent risk factors and establish predictive thresholds, to propose and validate optimized implant positioning strategies, and to evaluate the efficacy of these defined positional adjustments in mitigating perforation risk. The ultimate goal was to translate anatomic measurements and virtual planning data into a clinically applicable protocol for enhancing the safety and precision of implant placement in this anatomically complex region.

## Materials and methods

### Patient selection criteria

The study was approved by the Ethics Committee of the Affiliated Stomatological Hospital of Nanjing Medical University (PJ2023-113-001) and conducted in accordance with the tenets of the Declaration of Helsinki for research involving human subjects. Informed consent was obtained from all patients for being included in the study.

As a retrospective study, 297 patients who visited the Affiliated Stomatological Hospital of Nanjing Medical University between February 2023 and September 2024 and diagnosed with a missing maxillary premolar were screened. The inclusion criteria were listed as follows: (1) a single missing maxillary premolar, (2) a duration of tooth loss ≥ 3 months, (3) adjacent teeth without significant caries or periodontal disease, (4) adjacent teeth with a clearly identifiable and intact cementoenamel junction (CEJ) neighboring the edentulous space, and (5) the width of the alveolar crest in the edentulous area > 6 mm. The exclusion criteria were: (1) poor-quality CBCT images that precluded accurate measurement, (2) a history of trauma or surgery in the edentulous region that severely altered the native bone anatomy, (3) severe caries or the presence of restorations on adjacent teeth that obscured the identification of CEJ landmarks, (4) evidence of major maxillofacial deformities or pathologies in the maxilla or maxillary sinus, and (5) incomplete patient records or data. Then, a total of 252 patients were selected in the final analysis. This cohort comprised 153 (60.71%) females and 99 (39.29%) males, with a mean age of 46.73 ± 14.14 years (range: 17–81 years). All participants were of East Asian ethnicity.

### CBCT data acquisition

CBCT scans were acquired using the same device (NewTom 5G or NewTom VG; Verona, Italy) with the following standardized parameters: 110 kVp, 5.83 mA, field of view (FOV) = 18 × 16 cm, voxel size = 0.3 mm, and exposure time = 18 s. All the CBCT images were imported into ProPlan CMF (version 3.0, Materialise NV, Leuven, Belgium) for three-dimensional (3D) reconstruction of the maxilla and preliminary anatomical analysis of the alveolar bone. Subsequently, the data were transferred to SimPlant Pro (version 11.04, Materialise Dental, Leuven, Belgium) for virtual implant simulation, with angular and linear measurements performed at a software recording precision of 0.01° and 0.01 mm, respectively.

To ensure the measurement accuracy, three anatomical reference planes (axial, coronal, and sagittal) were re-established in the 3D reconstructed images to standardize head positioning, based on the methods proposed by Togashi et al. [16] and Cevidanes et al. [17] (Supplementary Fig. 1) and were listed as follows: axial plane (a plane passing through the left/right orbitale and the right porion); coronal plane (a plane passing through both porions and perpendicular to the axial plane); and sagittal plane (a plane passing through the anterior nasal spine (ANS) and gnathion (Gn), and perpendicular to the axial plane).

All the measurements and virtual implant placements were performed by the same investigator (Weihua Zhu) at two separate time points, three months apart, to assess intra-rater consistency. In cases of discrepancy, a senior clinician (Xiaomeng Song) reviewed and adjudicated the final results. Patient age and sex were also recorded during data collection.

### Virtual implant placement

The CEJ served as a consistent anatomical landmark delineating the boundary between the crown and root, with high visibility and reproducibility on CBCT scans, making it a reliable reference for implant trajectory planning [18]. Previous studies have shown that hard dental tissues can be used to construct stable occlusal reference planes [19], and adjacent teeth are commonly employed to assist in determining implant orientation [20, 21].

A localized crown reference plane was established using CEJ points from adjacent teeth, and a perpendicular line, referred to as the implant direction (ID) line, was constructed to represent the occlusal force vector of the missing tooth. Considering the functional and anatomical alignment between the long axis of the tooth and the direction of occlusal load transmission and bone remodeling [22], the method provided a biologically and biomechanically rational basis for implant trajectory design.

### Selection of implant dimensions

To simulate clinical conditions, a Nobel Active implant (Ref. 34131; Nobel Biocare AG, Zurich, Switzerland), with a cervical diameter of 4.3 mm, an apical diameter of 3.2 mm, and a length of 10 mm was selected for virtual placement. Although the use of implants with reduced dimensions might lower the risk of perforation to some extent, studies have indicated that short and narrow-diameter implants are associated with lower long-term survival rates and more stringent placement prerequisites compared to standard-sized implants [23, 24]. Furthermore, due to their inferior biomechanical performance, these smaller implants may be less suitable for the posterior region [25]. In contrast, the implant selected for the present study has been demonstrated by numerical analyses to offer excellent adaptability and primary stability, even in maxillary cases characterized by thin buccal bone plates or limited alveolar volume [26–28]. It is important to note that the selection of the 4.3-mm implant represents a methodological decision made to standardize the protocol in this study, rather than an indication of superior clinical performance.

### Selection of implant insertion site

**Fig. 1:**
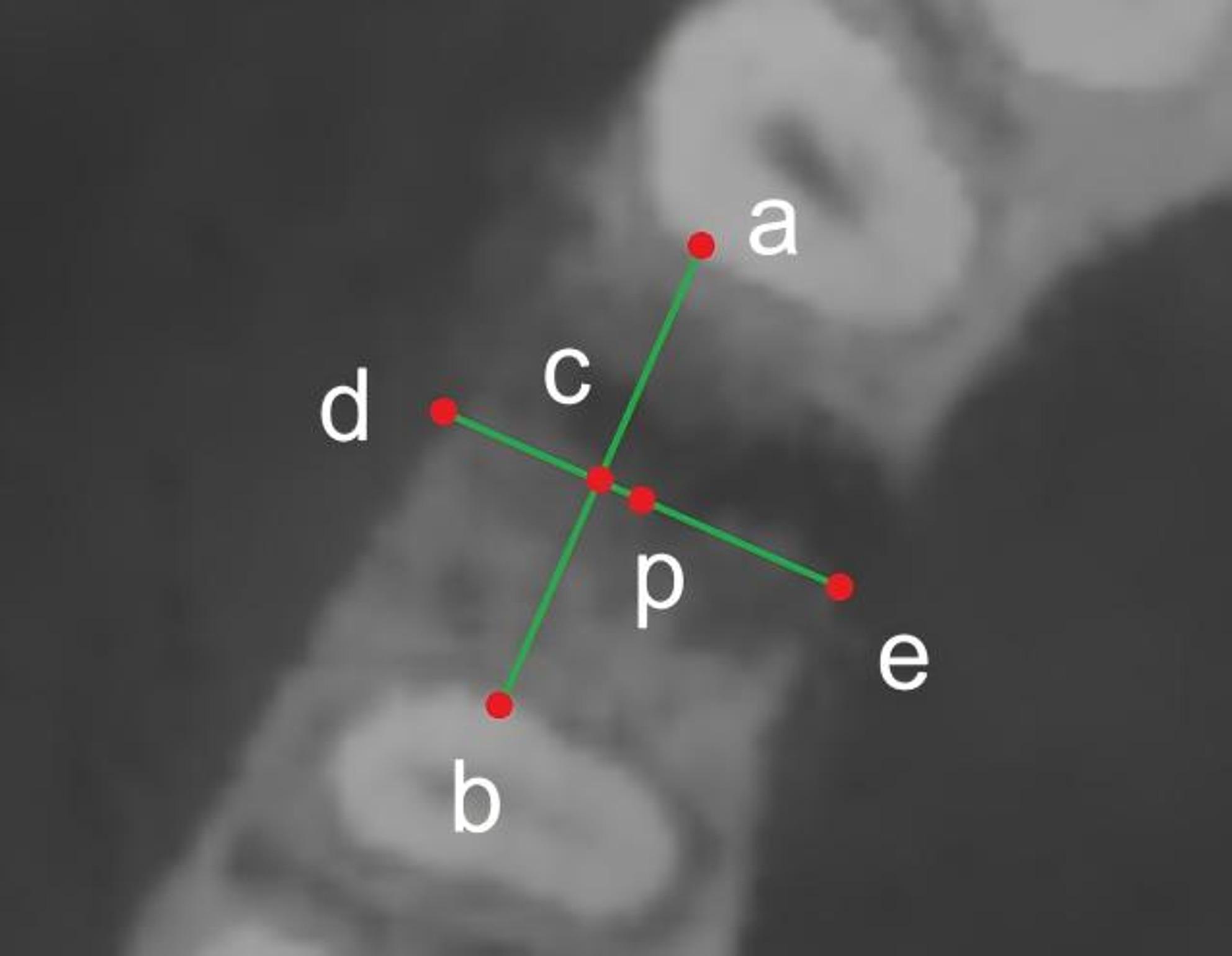
Schematic representation of how implant insertion sites were determined. Points a and b define the outermost contours of adjacent roots; c represents the midpoint of ab, and p represents the midpoint of de, representing the central insertion point

In order to preserve a safe distance between the implant and both the buccal and palatal cortical plates, as well as the roots of adjacent teeth, the CBCT viewing plane was initially oriented to the sagittal section and adjusted to 1 mm apical to the alveolar crest [29–31]. The most prominent distal contour of the mesial adjacent tooth root (point a) and the most prominent mesial contour of the distal adjacent tooth root (point b) were identified. A straight line (ab) was drawn to connect these two points, and its midpoint was defined as point c. From point c, a perpendicular line was extended toward the buccal (point d) and palatinal alveolar bone (point e), respectively. The midpoint of line de was designated as point p, representing the center of the residual alveolar bone in the edentulous region (Fig. 1).

### Determination of implant orientation

**Fig. 2:**
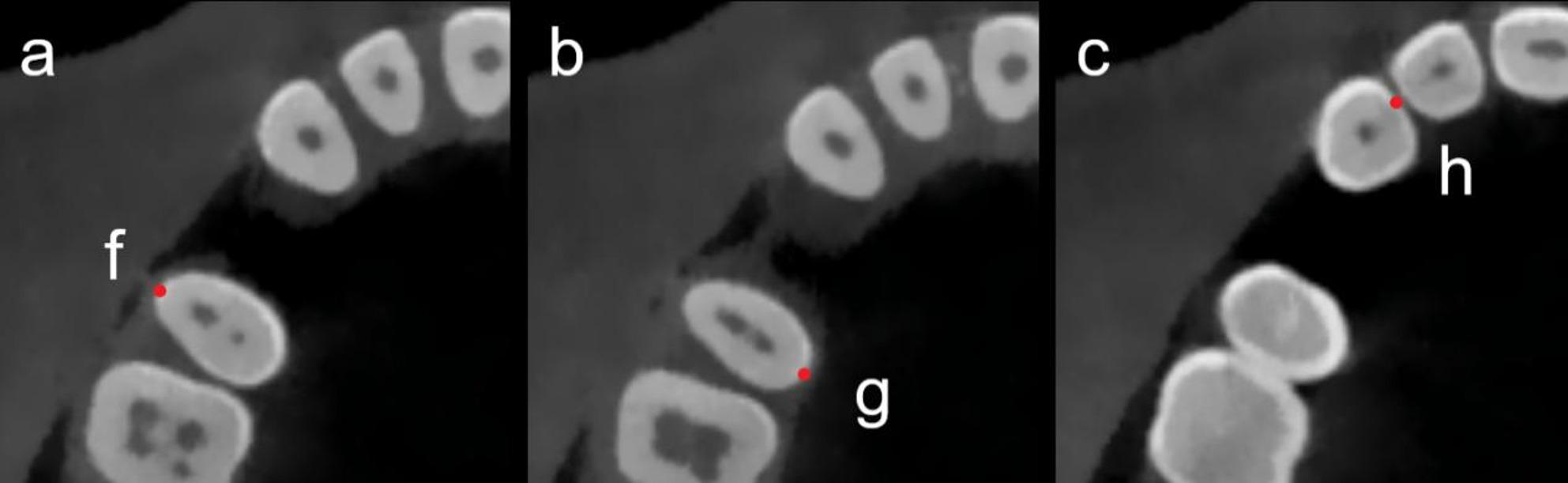
Identification of CEJ landmarks: **a** the buccal CEJ point (f) of the distal adjacent tooth, **b** the palatal CEJ point (g), and **c** mesial CEJ point (h) of the mesial adjacent tooth

**Fig. 3:**
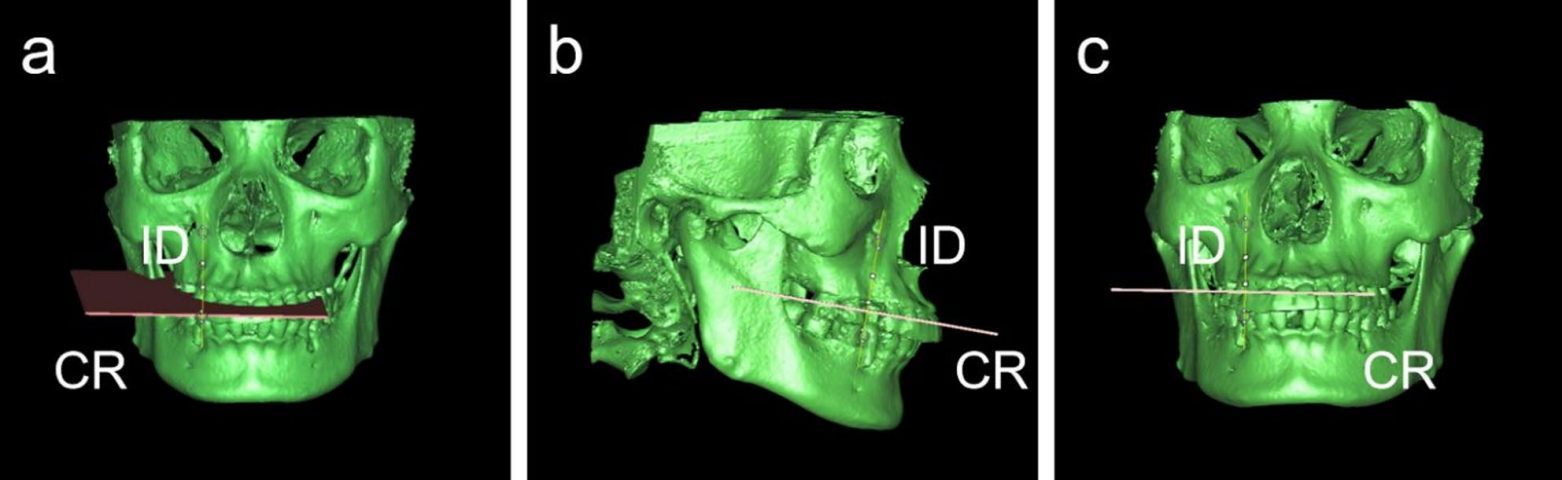
**a** Formation of the crown reference (CR) plane using points f, g, and h; **b**–**c** definition of the implant direction (ID) line as a perpendicular line through point p

Implant orientation was standardized using a crown reference (CR) plane constructed from CEJ landmarks of the adjacent teeth. Specifically, three reproducible anatomical points were identified: the most prominent buccal (f) and palatal (g) CEJ points of the distal adjacent tooth, and the most prominent mesial CEJ point (h) of the mesial adjacent tooth (Fig. 2). These landmarks were used to define the CR plane (Fig. 3a). The CR plane served as a geometric reference framework to ensure consistent determination of implant trajectory relative to the neighboring dentition and the restorative envelope [32]. A straight line passing through the planned implant entry point (p) and perpendicular to the CR plane was defined as the implant direction (ID) line, representing the simulated implant axis (Fig. 3b, c). BPP was assessed on multiplanar views and defined as any instance in which the simulated implant boundary extended beyond the outer cortical boundary of the buccal bone.

### Measurement of alveolar bone inclination angle

**Fig. 4:**
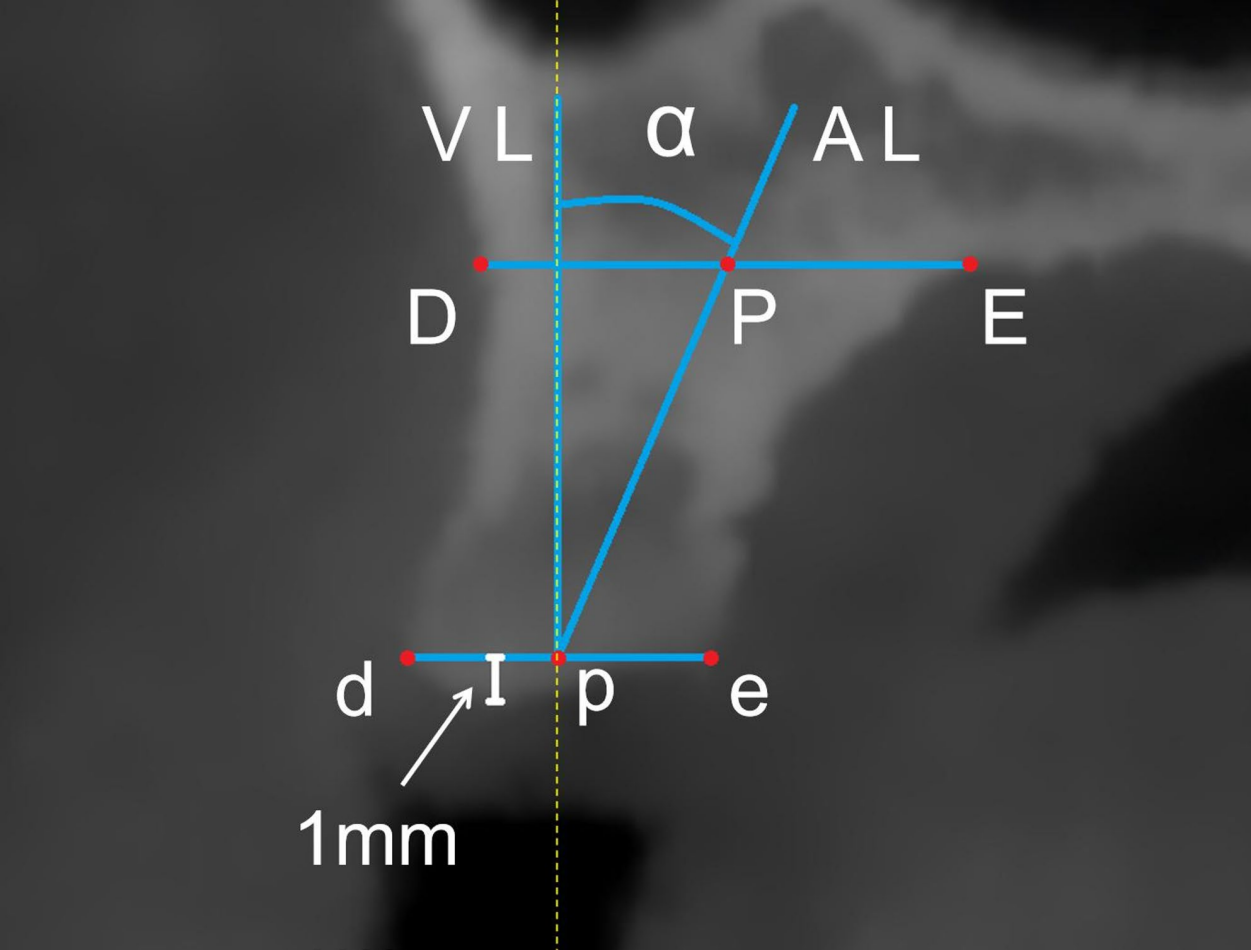
Measurement of alveolar bone inclination angle. DE represents the reference base line at the bottom of the alveolar bone. AL represents the long axis of the alveolar ridge. The angle α between AL and the vertical line (VL) indicates alveolar inclination

The observation plane was adjusted to a parasagittal-oblique section passing through the DE reference line and oriented perpendicular to the horizontal plane, enabling the assessment of residual alveolar bone morphology and inclination in the buccal to palatal direction. To standardize the measurement and minimize variability associated with crestal resorption, ABIA was consistently assessed at the level of the basal cortical boundary of the alveolar process. The original DE reference line was translated apically to the base of the alveolar bone, intersecting the buccal and palatal cortical plates at points D and E, respectively [33]. The line connecting these points was defined as the DE line. Point p was defined as the prosthetically planned implant entry point at the crestal level. From point p, the simulated implant direction line was extended apically along the planned implant trajectory until it intersected the basal cortical boundary at point P. Thus, the distance between p and P was determined by the anatomical extent of the available alveolar housing along the planned implant path and was not arbitrarily defined. A line connecting points p and P was defined as the alveolar bone long axis (AL line). The angle α formed between the AL line and the vertical reference line (VL line) represented the alveolar bone inclination angle (ABIA) in the buccopalatal direction (Fig. 4). Because the buccal and palatal cortical plates tend to diverge apically, ABIA may be sensitive to the vertical level at which it is measured. To reduce this potential variability, all measurements were referenced to the standardized basal cortical margin rather than the crestal height. This approach was selected because basal bone morphology is less affected by post-extraction remodeling and vertical ridge resorption, thereby providing a more stable anatomical landmark for angular assessment. A positive α value indicated that the AL line was positioned palatal to the VL line, whereas a negative value indicated a buccal position.

### Measurement of relative implant-to-bone angulation

**Fig. 5:**
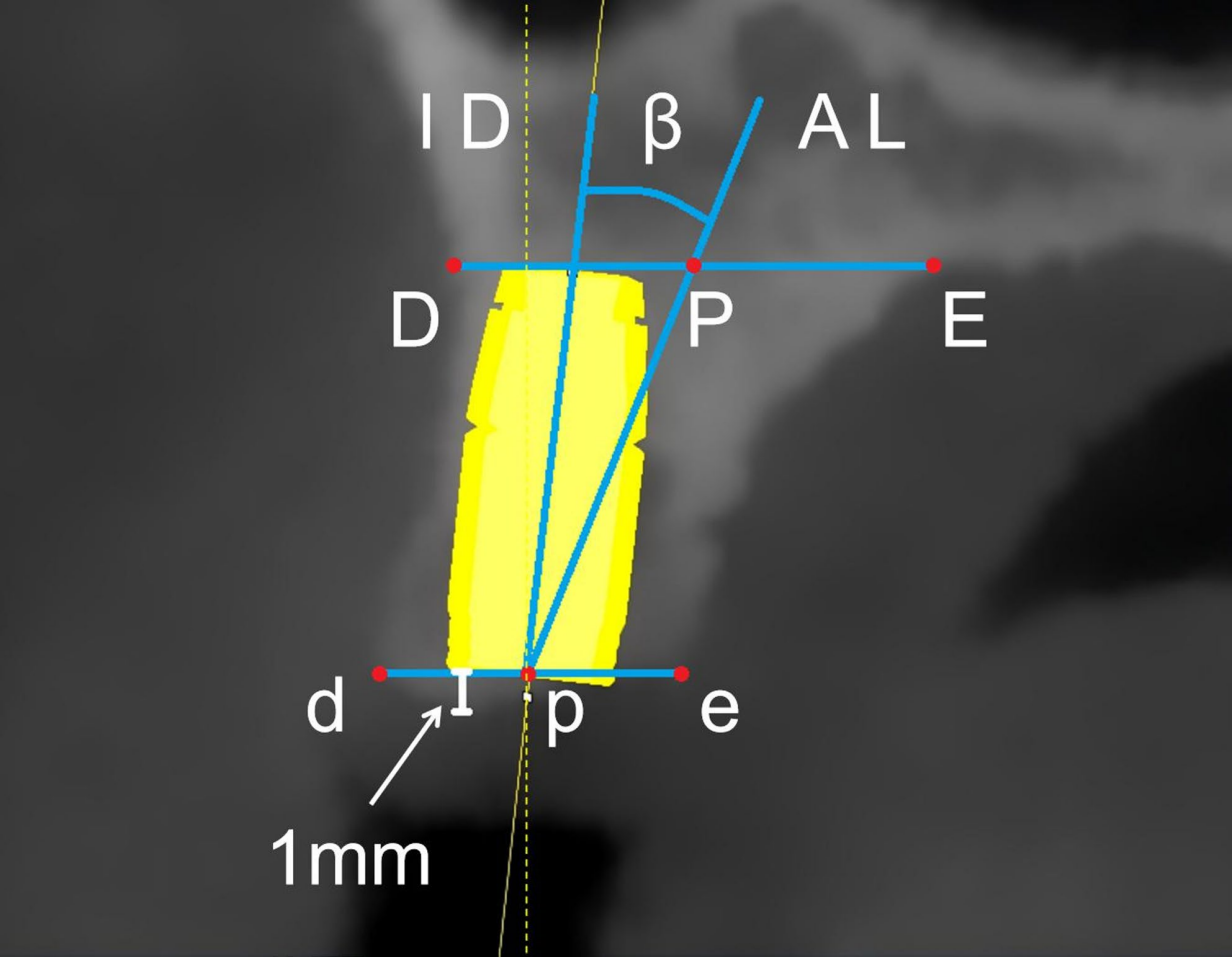
Measurement of relative implant-to-bone angulation. The implant direction (ID) line indicates the implant direction while the alveolar ridge (AL) line represents the alveolar ridge axis. The angle β quantifies the divergence between the implant axis and native bone inclination

The implant was virtually positioned along the ID line until the central axis of the implant neck coincided with point p, with the neck placed 1 mm apical to the alveolar crest in accordance with standard clinical protocol [29]. The implant’s central axis, automatically generated by the software, aligned with the ID line. The angle β between the ID line and the AL line was defined as the implant-to-bone angulation (IBA) (Fig. 5). A positive β value indicated that the ID line was positioned buccal to the AL line, whereas a negative value indicated a palatal position.

### Selection of alternative implant insertion sites

**Fig. 6:**
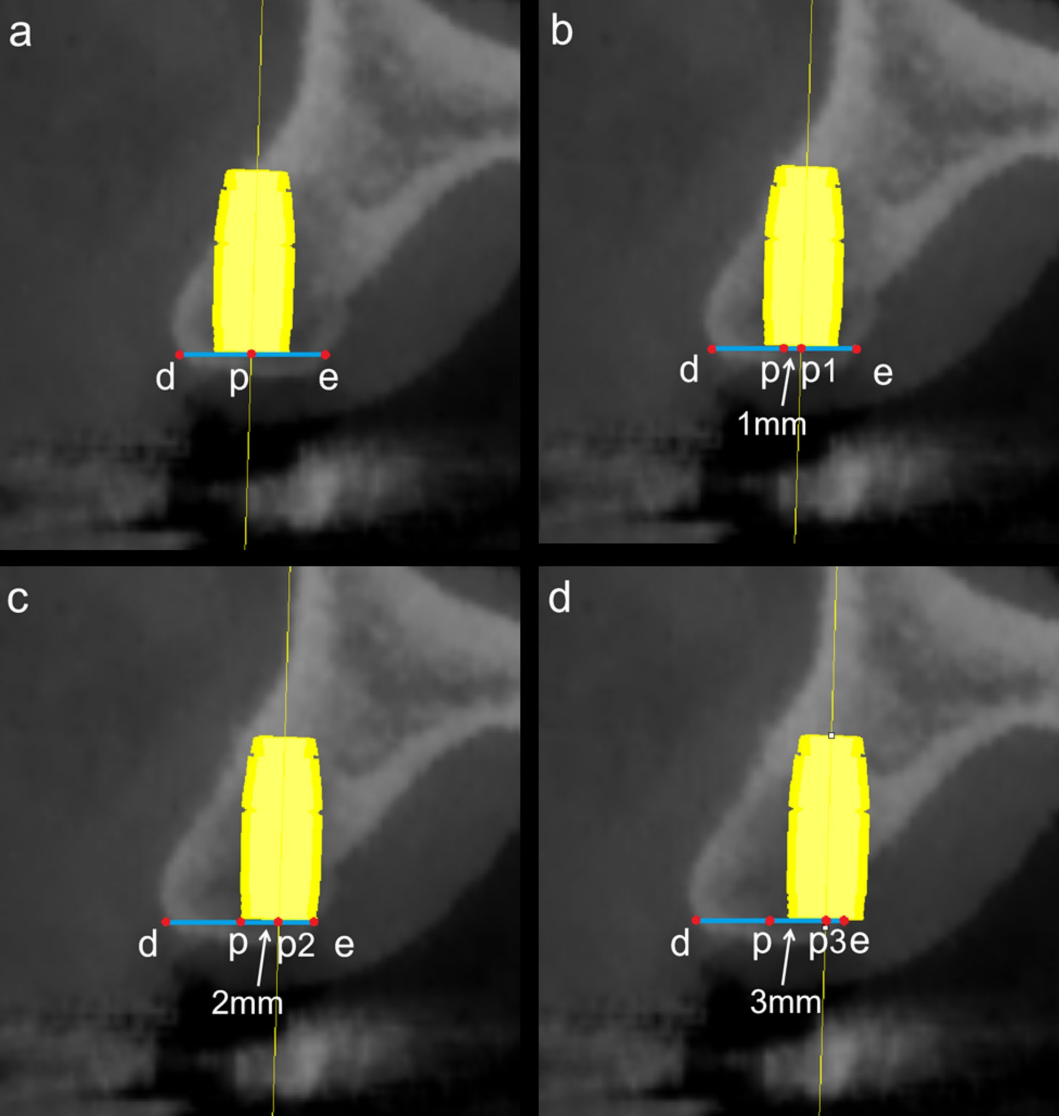
Simulation of four implant insertion sites: **a** central point p, **b** 1 mm palatal shift (p1), **c** 2 mm palatal shift (p2), and **d** 3 mm palatal shift (p3), all along the de reference axis

According to the findings of Lei et al. [34], positioning the implant slightly towards the palatal side may reduce the risk of buccal plate perforation. Guided by this rationale, four insertion positions were evaluated in the present study to access their effects on BPP incidence: the original central position (point p), and three additional positions (points p1, p2, and p3; located 1 mm, 2 mm, and 3 mm palatal to point p along the de axis, respectively) (Fig. 6).

### Classification of alveolar bone morphology

**Fig. 7:**
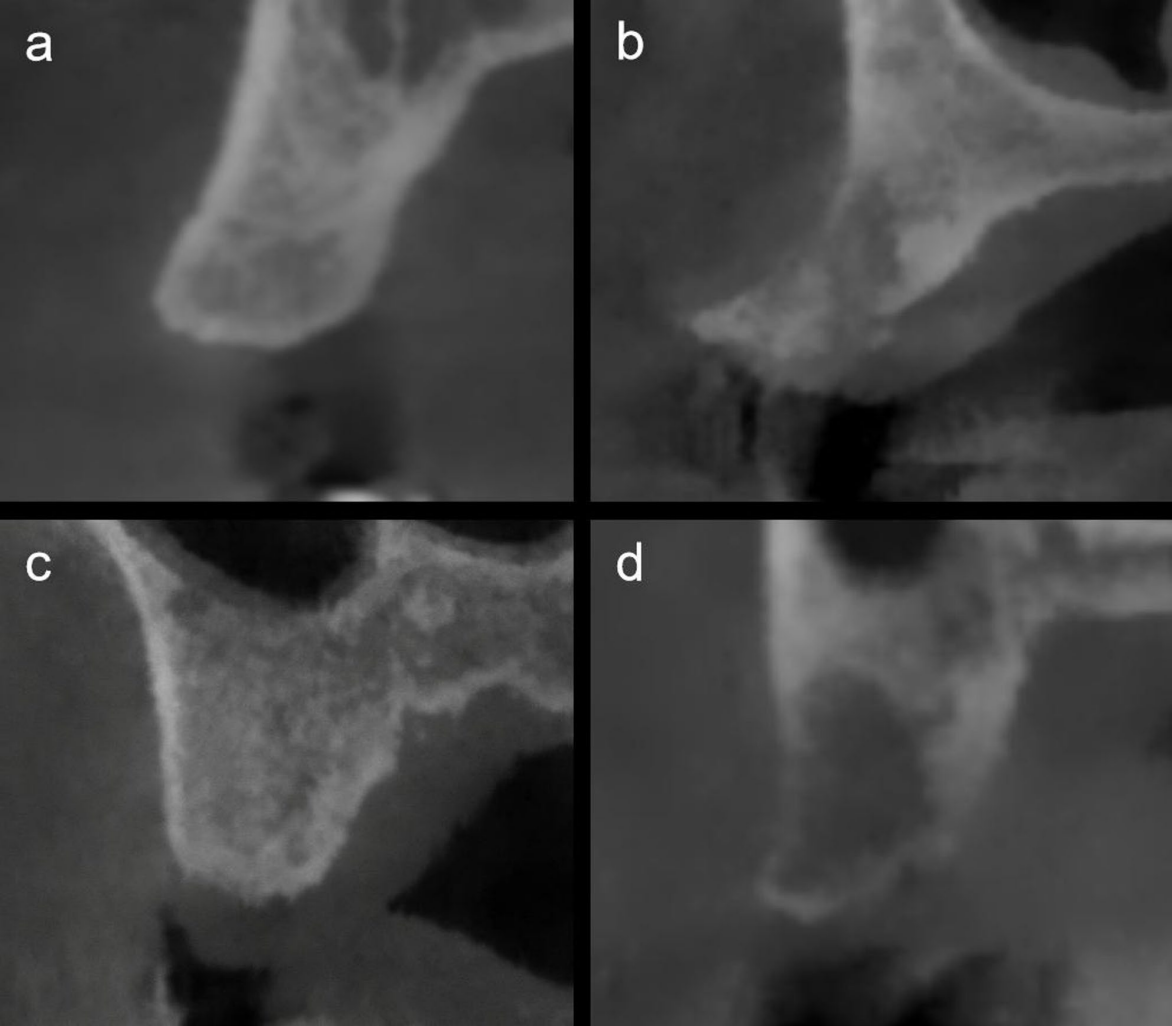
CBCT-based classification of alveolar bone morphology in the maxillary premolar region: **a** buccally projected-flat type; **b** buccally projected-concave type; **c** palatally retracted-flat type; **d** palatally retracted-concave type

Based on a comprehensive analysis of sagittal CBCT images from the included edentulous sites, the alveolar bone morphology in the maxillary premolar region was classified into four distinct types. This categorization reflects both the inherent inter-individual variability and the additional alterations resulting from post-extraction healing and remodeling:

- Buccally projected-flat type: buccally oriented alveolar ridge with a straight buccal cortical plate (Fig. 7a);
- Buccally projected-concave type: buccally oriented alveolar ridge with a concave buccal cortical plate (Fig. 7b);
- Palatally retracted-flat type: palatally oriented alveolar ridge with a straight buccal cortical plate (Fig. 7c);
- Palatally retracted-concave type: palatally oriented alveolar ridge with a concave buccal cortical plate (Fig. 7d).

### Statistical analysis

Categorical variables were presented as frequencies and percentages, whereas continuous variables were expressed as means ± standard deviations (SD). The Shapiro-Wilk (SW) test was first applied to assess the normality of continuous variables. Inter-measurement agreement for alveolar bone type and BPP occurrence between two measurements was evaluated using Cohen’s Kappa coefficient, while the intraclass correlation coefficient (ICC) was calculated to assess the repeatability of measurements for the ABIA and IBA.

For univariate analysis, Chi-squared tests were used to examine the associations between BPP incidence at point p and variables, including sex, age, alveolar bone type, and missing tooth position, as well as to compare BPP incidence among different implant insertion points (point p and palatal shifts of 1 mm, 2 mm, and 3 mm). Independent samples *t*-tests were performed to compare ABIA and IBA between the BPP and non-BPP groups. Variables with potential significance were further entered into a multivariate logistic regression model to identify independent predictors of BPP at point p. Receiver operating characteristic (ROC) analysis with Youden’s J statistic (J index) was performed to determine the optimal cutoff values of IBA and ABIA corresponding to the threshold angles for predicting BPP. In addition, perforation rates at four simulated insertion points (p, p1, p2, and p3) were analyzed and compared across different anatomical and clinical variables. Multivariate logistic regression analyses were subsequently performed to identify the independent predictors of perforation at each insertion point. All the statistical analyses were performed using SPSS software, version 25.0 (IBM Corp., Armonk, NY, USA), and *P* value < 0.05 (2-tailed) was considered statistically significant.

## Results

### Consistency and reproducibility of imaging measurements

Cohen’s Kappa values for two independent measurements of alveolar bone type, BPP occurrence at point p, and its palatal variants (p1–p3) ranged from 0.852 to 0.906, indicating a high level of inter-measurement agreement. The ICC values for ABIA and IBA were 0.901 and 0.909, respectively, confirming the excellent intra-observer reliability and repeatability of the imaging methodology employed in the present study (Supplementary Table 1).

### Epidemiological characteristics of 252 patients with a single maxillary premolar loss

As summarized in Table 1, a total of 252 patients who met the inclusion criteria were included in the final analysis. Patients age ranged from 17 to 81 years, with a mean age of 46.73 ± 14.14 years. The cohort comprised 153 females (60.71%) and 99 males (39.29%). Regarding the missing tooth position, 98 patients (38.89%) were missing the maxillary first premolar, whereas 154 patients (61.11%) were missing the maxillary second premolar. In terms of alveolar bone type, the most common type was the buccally projected-flat type (102 cases, 40.48%), followed by the buccally projected-concave type (62 cases, 24.60%), the palatally retracted-flat type (65 cases, 25.79%), and palatally retracted-concave type (23 cases, 9.13%).

**Table 1:** Comparisons of clinical and imaging variables between the BPP and non-BPP groups

Variable	Overall(*N* = 252)	Non-perforation group (*n* = 197)	Perforation group (*n* = 55)	t/χ^2^ value	*P* value
Age (years)	46.73 ± 14.14 (17–81)	46.72 ± 13.98	46.78 ± 14.95	-0.031	0.976^a^
Sex				5.643	0.019*
Male	99 (39.29%)	85 (85.86%)	14 (14.14%)		
Female	153 (60.71%)	112 (68.71%)	41 (31.29%)		
Alveolar bone type				44.828	< 0.001*
Buccally projected – flat	102 (40.48%)	80 (78.43%)	22 (21.57%)		
Buccally projected – concave	62 (24.60%)	32 (51.61%)	30 (48.39%)		
Palatally retracted – flat	65 (25.79%)	65 (100.00%)	0 (0.00%)		
Palatally retracted – concave	23 (9.13%)	20 (86.96%)	3 (13.04%)		
Tooth position				15.564	< 0.001*
First premolar	98 (38.89%)	64 (65.31%)	34 (34.69%)		
Second premolar	154 (61.11%)	133 (86.36%)	21 (13.64%)		
ABIA (°)	19.38 ± 8.50	17.03 ± 7.30	27.82 ± 7.06	-9.765	< 0.001^a^*
IBA (°)	19.22 ± 9.43	16.55 ± 8.02	28.77 ± 7.83	-10.036	< 0.001^a^*

Abbreviations: ABIA, Alveolar bone inclination angle; IBA, Implant-to-bone angle

^a^Independent samples *t*-test

^*^Statistically significant (*P *< 0.05)

### Risk factors associated with BPP at point p

Patients were divided into two groups based on the occurrence of BPP at the initial insertion point (p): the BPP group (*n* = 55) and the non-BPP group (*n* = 197). Chi-squared tests and independent t-tests were used to compare epidemiological and 3D radiographic characteristics between the two groups (Table 1). Sex, implant position, alveolar bone type, ABIA and IBA were significantly associated with the incidence of BPP (*P* < 0.05). A single negative ABIA value of -4.17° was observed in 1 case (0.40%), which was classified as palatally retracted-flat alveolar bone morphology. Negative IBA values were identified in 7 cases (2.78%), with the minimum value being − 4.42°. Among these cases, 6 were associated with palatally retracted-flat alveolar bone morphology and 1 was associated with buccally projected-concave alveolar bone morphology. Importantly, no BPP events were observed in cases presenting with negative ABIA or IBA values.

When framed by the frequency of perforation, the BPP rate was higher in females (31.29%) than in males (14.14%), and markedly higher at first premolar sites (34.69%) compared to second premolar sites (13.64%). Among the alveolar bone types, the buccally projected-concave type presented the highest perforation rate (48.39%). However, age was not significantly associated with the incidence of BPP (*P* > 0.05).

Multivariate logistic regression analysis was conducted (Table 2), with BPP status as the dependent variable (1 = BPP, 0 = non-BPP). All variables with significant differences in the univariate analysis, along with age as a potential confounder, were entered as independent variables. Considering the instability of the regression models due to complete separation caused by zero BPP events in the palatally retracted-flat bone morphology (Table 1), the buccally projected-flat and palatally retracted-flat types were regrouped into a “flat” category, whereas the buccally projected-concave and palatally retracted-concave types were regrouped into a “concave” category based on the morphological characteristics of the alveolar bone. Collinearity analysis showed variance inflation factor values of 2.186 for both ABIA and IBA, indicating no significant multicollinearity (Supplementary Table 2). Regarding risk factors, female patients had a significantly higher risk of BPP than males (OR = 4.466, 95%CI = 1.696–11.758, *P* = 0.002). In addition, the risk of BPP at the second premolar site was significantly lower than that at the first premolar site (OR = 0.215, 95% CI = 0.083–0.559, *P* = 0.002). In terms of alveolar bone morphology, the concave type was associated with a significantly higher risk of BPP compared with the flat type (OR = 5.699, 95% CI = 2.208–14.713, *P* < 0.001). Moreover, each 1° increase in ABIA was associated with a 19.1% increase in BPP risk (OR = 1.191, 95% CI = 1.091–1.300, *P* < 0.001) and that each 1° increase in IBA was associated with a 12.9% increase in risk (OR = 1.129, 95% CI = 1.047–1.217, *P* = 0.002).

**Table 2:** Multivariate logistic regression identifying independent predictors of BPP

Variable		OR	95%CI		*P* value
			Lower	Upper	
Age (years)		1.011	0.980	1.043	0.488
Sex	Male				
	Female	4.466	1.696	11.758	0.002*
Tooth position	First Premolar				
	Second Premolar	0.215	0.083	0.559	0.002*
Alveolar bone type	Flat^a^				
	Concave^b^	5.699	2.208	14.713	< 0.001*
ABIA		1.191	1.091	1.300	< 0.001*
IBA		1.129	1.047	1.217	0.002*

*Abbreviations*: *BPP* Buccal plate perforation, *ABIA* Alveolar bone inclination angle, *IBA* Implant-to-bone angle

^a^including buccally projected-flat and palatally retracted-flat types, ^b^ including buccally projected-concave and palatally retracted-concave types

^*^Statistically significant (*P* < 0.05)

### ROC analysis and predictive performance

**Fig. 8:**
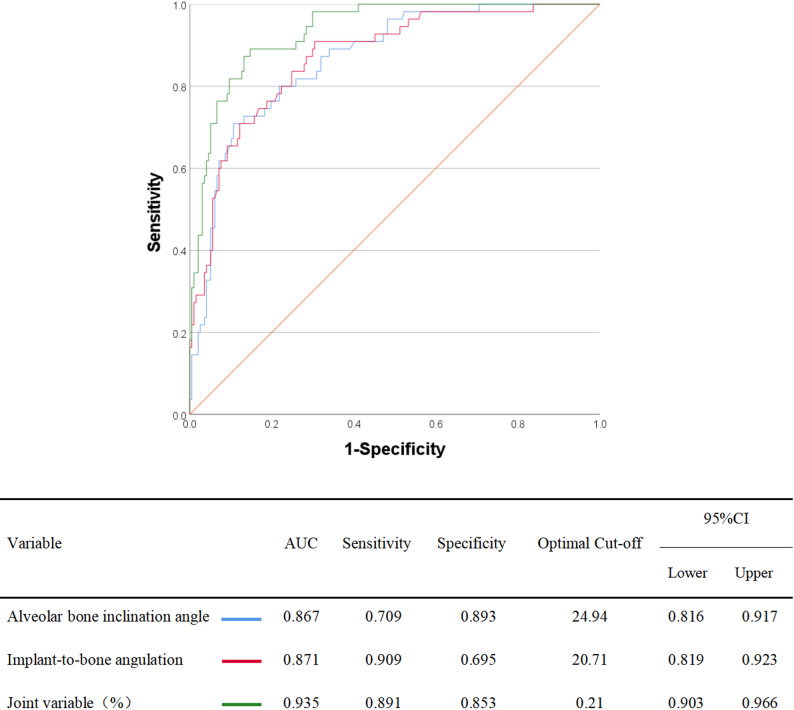
ROC curves of alveolar bone inclination angle, implant-to-bone angulation and joint variable in the predicting of buccal plate perforation

 As shown in Fig. 8, both ABIA and IBA exhibited strong predictive performance for BPP in the maxillary premolar region. ABIA yielded an AUC of 0.867 (95%CI = 0.816–0.917), with an optimal cutoff value of 24.94°, a sensitivity of 0.709, and a specificity of 0.893. IBA demonstrated an AUC of 0.871 (95%CI = 0.819–0.923), with an optimal cutoff value of 20.71°, a sensitivity of 0.909, and a specificity of 0.695. When the predicted probability values from the logistic regression model (Table 3) were combined into a composite variable, the resulting ROC curve showed an improved AUC of 0.935 (95%CI = 0.903–0.966), with an optimal-cut off value of 0.21, a sensitivity of 0.891, and a specificity of 0.853.

**Table 3:** Buccal and palatal perforation rates at different implant insertion point across various anatomical factors.

Variable		p	p1	p2	p3
Tooth position					
First premolar	Non-perforation	64(65.31%)	82(83.67%)	49(50.00%)	13(13.27%)
(n = 98)	Buccal Perforation	34(34.69%)	14(14.29%)	11(11.22%)	2(2.04%)
	Palatal Perforation	0(0.00%)	2(2.04%)	38(38.78%)	83(84.69%)
Second premolar	Non-perforation	133(86.36%)	148(96.10%)	123(79.87%)	47(30.52%)
(n = 154)	Buccal Perforation	21(13.64%)	4(2.60%)	2(1.30%)	0(0.00%)
	Palatal Perforation	0(0.00%)	2(1.30%)	29(18.83%)	107(69.48%)
Bone morphology					
Flat type^a^	Non-perforation	145(86.83%)	155(92.81%)	114(68.26%)	35(20.96%)
(n = 167)	Buccal Perforation	22(13.17%)	8(4.79%)	4(2.40%)	0(0.00%)
	Palatal Perforation	0(0.00%)	4(2.40%)	49(29.34%)	132(79.04%)
Concave type^b^	Non-perforation	52(61.18%)	75(88.24%)	58(68.24%)	25(29.41%)
(n = 85)	Buccal Perforation	33(38.82%)	10(11.76%)	9(10.59%)	2(2.35%)
	Palatal Perforation	0(0.00%)	0(0.00%)	18(21.18%)	58(68.24%)
ABIA					
< 24.94°	Non-perforation	176(91.67%)	185(96.35%)	133(69.27%)	44(22.92%)
(n = 192)	Buccal Perforation	16(8.33%)	4(2.08%)	3(1.56%)	1(0.52%)
	Palatal Perforation	0(0.00%)	3(1.56%)	56(29.17%)	147(76.56%)
≥ 24.94°	Non-perforation	21(35.00%)	45(75.00%)	39(65.00%)	16(26.67%)
(n = 60)	Buccal Perforation	39(65.00%)	14(23.33%)	10(16.67%)	1(1.67%)
	Palatal Perforation	0(0.00%)	1(1.67%)	11(18.33%)	43(71.67%)
IBA					
< 20.71°	Non-perforation	137(96.48%)	138(97.18%)	95(66.90%)	34(23.94%)
(n = 142)	Buccal Perforation	5(3.52%)	0(0.00%)	0(0.00%)	1(0.70%)
	Palatal Perforation	0(0.00%)	4(2.82%)	47(33.10%)	107(75.35%)
> 20.71°	Non-perforation	60(54.55%)	92(83.64%)	77(70.00%)	26(23.64%)
(n = 110)	Buccal Perforation	50(45.45%)	18(16.36%)	13(11.82%)	1(0.91%)
	Palatal Perforation	0(0.00%)	0(0.00%)	20(18.18%)	83(75.45%)

*Abbreviations*: *BPP* Buccal plate perforation, *ABIA* Alveolar bone inclination angle, *IBA* Implant-to-bone angle

^a^including buccally projected-flat and palatally retracted-flat types, ^b^ including buccally projected-concave and palatally retracted-concave types

### Effect of different palatal shift distance from point p on perforation rates

The type and incidence of perforation varied markedly with palatal displacement from the original implant insertion point (p) (Table 3). At point p, the incidence of BPP was 34.69% in the first premolar region and 13.64% in the second premolar region, with no cases of palatal perforation observed. A 1-mm palatal shift (p1) significantly reduced BPP rates to 14.29% and 2.60%, respectively, while maintaining low palatal perforation rates of 2.04% and 1.30%. As the implant position was shifted further palatally (p2, p3), BPP continued to decrease, whereas the incidence of palatal perforation increased, reaching the highest rates at a 3-mm shift (p3) of 84.69% and 69.48%.

For flat bone morphology, the BPP rate was 13.17% at point p, whereas for concave bone morphology, the rate was higher at 38.82%. At point p1, BPP decreased to 4.79% and 11.76%, respectively, with low palatal perforation rates (2.40% and 0%). However, further palatal shifts (p2, p3) led to an increase in palatal perforation, with rates rising to 79.04% and 68.24%, respectively. When ABIA and IBA were below the cut off values of 24.94° and 20.71°, respectively, BPP rates remained low. In contrast, when ABIA ≥ 24.94° and IBA ≥ 20.71°, BPP rates increased significantly at p1 (23.33% and 16.36%, respectively), accompanied by an increase in palatal perforation.

Table 4 presents the multivariate logistic regression analyses identifying independent predictors of BPP at the four simulated insertion points (p, p1, p2, and p3). Age was not significantly associated with BPP at any insertion point (*P* > 0.05). Female sex was significantly associated with an increased risk of BPP at p (OR = 3.013, 95% CI = 1.191–7.621, *P* = 0.020), p2 (OR = 2.377, 95% CI = 1.265–4.469, *P* = 0.007), and p3 (OR = 3.775, 95% CI = 1.992–7.163, *P* < 0.001), whereas no significant association was observed at p1 (*P* = 0.263). Tooth position demonstrated a consistent protective effect of the second premolar relative to the first premolar across all insertion points, with statistically significant associations at point p (OR = 0.211, 95% CI = 0.081–0.551, *P* = 0.001), p1 (OR = 0.266, 95% CI = 0.091–0.778, *P* = 0.016), p2 (OR = 0.173, 95% CI = 0.091–0.328, *P* < 0.001), and p3 (OR = 0.286, 95% CI =0.137-0.595, *P* = 0.001). For alveolar bone morphology, the concave type was significantly associated with an increased risk of BPP at point p (OR = 5.631, 95% CI = 2.170-14.612, *P* < 0.001), whereas this association was not statistically significant at p1, p2, or p3 (*P* > 0.05). Regarding angular parameters, ABIA ≥ 24.94° was strongly associated with an increased risk of BPP at p (OR = 13.594, 95% CI = 5.363–34.460, *P* < 0.001) and remained significant at p1 (OR = 5.028, 95% CI = 1.691–14.954, *P* = 0.004), but not at p2 or p3. Similarly, IBA ≥ 20.71° was significantly associated with BPP at points p (OR = 6.864, 95% CI = 2.399–34.460, *P* < 0.001) and p2 (OR = 0.389, 95% CI = 0.191–0.790, *P* = 0.009), whereas no significant association was observed at point p1 or p3.

**Table 4:** Multivariate logistic regression identifying independent predictors of buccal plate perforation across different insertion points.

Variable		p		p1		p2		p3	
		OR (95%CI)	*P* value	OR (95%CI)	*P* value	OR (95%CI)	*P* value	OR (95%CI)	*P* value
Age (years)		1.013(0.983,1.045)	0.393	0.999(0.965,1.033)	0.935	1.004(0.983,1.025)	0.724	0.992(0.970,1.015)	0.497
Sex	Male								
	Female	3.013(1.191,7.621)	0.020*	1.884(0.621,5.719)	0.263	2.377(1.265,4.469)	0.007*	3.775(1.992,7.163)	< 0.001*
Tooth position	First Premolar								
	Second Premolar	0.211(0.081,0.551)	0.001*	0.266(0.091,0.778)	0.016*	0.173(0.091,0.328)	< 0.001*	0.286(0.137,0.595)	0.001*
Alveolar bone type	Flat type^a^								
	Concave type^b^	5.631(2.170,14.612)	< 0.001*	1.310(0.470,3.654)	0.606	1.464(0.771,2.781)	0.244	0.759(0.389,1.480)	0.418
ABIA (°)	< 24.94°								
	≥ 24.94°	13.594(5.363,34.460)	< 0.001*	5.028(1.691,14.954)	0.004*	1.336(0.620,2.881)	0.459	0.868(0.385,1.958)	0.733
IBA (°)	< 20.71°								
	≥ 20.71°	6.864(2.399,34.460)	< 0.001*	2.340(0.650,8.426)	0.193	0.389(0.191,0.790)	0.009*	0.787(0.383,1.617)	0.514

*Abbreviations*: *BPP* Buccal plate perforation, *ABIA* Alveolar bone inclination angle, *IBA* Implant-to-bone angle

^a^including buccally projected-flat and palatally retracted-flat types, ^b^including buccally projected-concave and palatally retracted-concave types

^*^Statistically significant (*P* < 0.05)

## Discussion

The maxillary premolar region presents significant challenges for implant placement due to its anatomical complexity and limited bone volume [6, 7]. Previous studies have largely overlooked the substantial post-extraction bone remodeling that occurs during the healing phase, which can significantly affect implant planning [13–15]. This study, using CBCT data from patients at least three months after tooth extraction, aimed to quantitatively evaluate risk factors for BPP and to develop clinically applicable strategies for optimizing implant positioning.

BPP is not merely a radiographic finding but may have important biological and esthetic implications. Although limited apical cortical exposure does not necessarily compromise short-term implant survival, disruption of the buccal plate has been associated with increased marginal bone remodeling, especially in patients with thin buccal bone or delicate soft-tissue biotypes. Loss of buccal cortical integrity may impair vascular supply, contribute to soft-tissue recession, and increase the risk of thread exposure, plaque accumulation, and peri-implant inflammation [35, 36]. In esthetically sensitive areas, even minor buccal deficiencies may lead to long-term mucosal instability. Therefore, although BPP does not invariably result in implant failure, current evidence suggests that it represents a clinically relevant event that may adversely affect biological stability and esthetic outcomes over time.

In this study, the implant axis was determined using a crown reference (CR) plane constructed from the cementoenamel junctions (CEJ) of adjacent teeth [7, 18, 21], providing a prosthetically driven and anatomically reproducible reference framework derived from stable hard-tissue landmarks [32]. Unlike ridge-centered approaches that may be distorted by post-extraction remodeling, CEJ-based alignment preserves the original tooth axis, reduces operator-dependent variability, enhances inter-case comparability, and aligns virtual planning with restorative principles. The CEJ was prioritized over occlusal landmarks (e.g., cusp tips), as the latter are susceptible to wear or caries, and inherent differences in both height and volume between buccal and palatal cusps in maxillary premolars could introduce directional bias into the reference plane [37, 38]. Alternative methods, such as statistical shape modeling, have been proposed for automated trajectory optimization but currently have limited routine clinical application due to requirements for large datasets and specialized software [39]. In contrast, the CR-plane method offers a clinically accessible and reproducible framework suitable for standard CBCT-based preoperative assessment.

Multivariate analysis confirmed several patient- and site-specific risk factors for BPP. Female sex was identified as a significant risk factor in the maxillary premolar region, consistent with previous findings of thinner buccal bone and more pronounced alveolar concavity in females [5, 6], and extending similar observations from the anterior maxilla [12] to the premolar region. Tooth position also significantly influenced BPP risk, with first premolars exhibiting higher perforation rates than second premolars, which may be attributable to thinner bone and more complex ridge morphology [12, 40, 41]. These findings underscore the need for individualized preoperative planning, which can be further enhanced by digital surgical guides to improve placement precision [12].

In our cohort, the incidence of BPP at the ridge-centered position (point p) was 34.69% for first premolars and 13.64% for second premolars, which is lower than the 47.6% reported by Alqutaibi et al. [6]. This discrepancy may reflect methodological differences between the two studies. First, our simulations were based on CBCT scans obtained after a minimum three-month post-extraction healing period, whereas Alqutaibi et al. analyzed dentate sites where post-extraction alveolar remodeling [15] would not have occurred. Second, the implant placement methodology differed. Alqutaibi et al. aligned the virtual implant with the long axis of the natural tooth and positioned the platform at the buccal crestal level, whereas our study employed a prosthetically driven approach using a CEJ-based crown reference plane, with the platform placed 1 mm apical to the crest without buccal bias. Additional factors, including population differences (East Asian vs. Middle Eastern) and potential variations in perforation classification criteria, may also have contributed.

Alveolar bone morphology was identified as a significant predictor of BPP at the original insertion point (p), with concave bone types exhibiting a higher perforation risk than flat types. This finding likely reflects the greater ridge angulation and reduced buccal bone support characteristic of concave morphologies, which increase the risk of cortical breach when implants follow a prosthetically driven axis. Previous CBCT-based studies have similarly reported elevated BPP risk in premolar and canine regions with pronounced ridge inclination or buccal concavity [6, 12, 13]. Notably, this association was no longer statistically significant at the shifted insertion points (p1-p3), suggesting that minor palatal repositioning may mitigate the morphological disadvantage of concave ridges. These findings underscore the importance of three-dimensional ridge assessment [42], as perforation risk reflects not only surface contour but also the overall buccolingual bone distribution along the implant trajectory [43]. In contrast, the more uniform cortical profile of flat-type ridges may provide more predictable bone support when minor positional adjustments are made. Taken together, these results indicate that although concave morphology is a risk factor at conventional ridge-centered positions, its adverse effects can be substantially attenuated through slight palatal displacement of the implant.

Our results align with previous CBCT-based studies emphasizing the importance of three-dimensional ridge morphology and implant-bone angulation in predicting perforation risk [14, 44]. Consistent with these findings, angular parameters such as ABIA and IBA provided more accurate risk prediction than linear thickness measurements alone. Extending prior research by focusing on healed post-extraction sites, this study established quantitative cutoff values for more precise risk stratification: ABIA > 24.94° and IBA > 20.71° were identified as key predictors of perforation risk [13, 43], offering valuable reference points for preoperative assessment and implant trajectory optimization. However, it is important to note that these cutoff values are specific to the sample in this study, and their applicability to other populations or clinical settings may require further validation.

Regarding measurement methodology, although residual ridge height varies among individuals and could theoretically influence angular measurements due to apical divergence of cortical plates, ABIA was consistently referenced to the basal cortical boundary, ensuring measurement at a standardized anatomical level. While differences in ridge height may affect the absolute length of the implant trajectory within the alveolar housing, they do not alter the geometric definition of ABIA as the angle between the AL line and the vertical reference line. Therefore, the potential impact of vertical bone height variability on study outcomes is considered limited.

A key finding of this study was the effectiveness of a 1 mm palatal shift in reducing BPP incidence, achieving a substantial reduction in risk while maintaining a low rate of palatal perforation. This benefit was observed across various anatomical features, with particular effectiveness in complex morphologies such as concave bone types. However, despite this overall reduction in risk, tooth position and ABIA remained significant independent predictors of BPP at point p1. First premolars exhibited a higher risk than second premolars, and elevated ABIA values were strongly associated with increased perforation risk. In contrast, larger shifts (2 mm and 3 mm), although further reducing BPP, led to a marked increase in palatal perforation, especially at point p3. These findings suggest that a 1 mm palatal shift offers an optimal balance, effectively mitigating BPP risk while minimizing palatal compromise, particularly in patients with high ABIA or IBA or with challenging anatomical features such as first premolars and concave ridge morphology.

This study has several limitations. Its single-center, retrospective design may introduce selection bias, and known risk factors for buccal plate perforation, such as implant system choice and soft tissue biotype, were not stratified in the analysis [7, 13, 43]. Additionally, the CR plane was introduced as an anatomical standardization tool. While this method represents a novel approach, it is grounded in principles from restorative dentistry and digital planning. Furthermore, occlusal contacts and antagonist dentition were not incorporated into the current CBCT-based model; therefore, the approach does not account for patient-specific functional loading conditions. As a virtual simulation, the findings lack clinical validation. Nevertheless, the predictive thresholds and the 1 mm palatal offset strategy established here offer an important framework for preoperative risk assessment. The most precise and reproducible implementation of this framework can be achieved through guided surgery systems, such as CAD/CAM static surgical guides or dynamic navigation [45, 46], which are designed for submillimeter accuracy [47, 48]. Furthermore, integrating these evidence-based parameters into planning software provides a foundation for advancing guided systems from basic execution tools to intelligent systems capable of automated risk stratification and trajectory optimization. Since the primary goal of this study was to identify anatomical risk factors rather than to develop or validate a clinical prediction model, internal validation procedures such as bootstrap correction and formal calibration assessment were not performed. Future prospective, multi-center studies incorporating these technologies are needed to clinically validate and refine the proposed protocol.

## Conclusion

In conclusion, female sex, first premolar position, and concave alveolar morphology were identified as significant risk factors for BPP in the maxillary premolar region. Threshold values for ABIA (> 24.94°) and IBA (> 20.71°) were associated with a significantly increased risk of perforation, suggesting their potential utility as quantitative indicators in preoperative planning. Additionally, a 1 mm palatal shift from the center of the residual alveolar ridge was identified as a potential strategy for minimizing perforation risk in virtual simulations. Although the CR-plane-based measurements provide a structured framework for CBCT-guided risk assessment, it should be noted that this study is based on virtual simulations, and further prospective validation across broader anatomical variations is required. Incorporating these parameters into virtual surgical planning may provide a more objective approach to optimizing implant trajectories and enhancing surgical safety, although clinical validation is necessary before these methods can be widely applied.

## Supplementary Information

Supplementary Material 1.

## Data Availability

All data generated or analysed during this study are included in this published article and its supplementary information files.
