# Supplementary material for: CBCT-based virtual surgical planning for predicting risk factors and preventive strategies of buccal plate perforation in maxillary premolar implant placement
Source: Head Face Med. 2026 Mar 18;22:46. doi: 10.1186/s13005-026-00611-3 (PMC13113480; doi:10.1186/s13005-026-00611-3)
Supplement: Supplementary file 1 — Supplementary Material 1. [file 13005_2026_611_MOESM1_ESM.docx]

**Supplementary Table 1.** Intra-observer agreement for categorical and continuous variables, expressed as Cohen’s kappa or intraclass correlation coefficient.

| Variable | Inter-observer agreement |
| --- | --- |
| Alveolar bone type | 0.864^a^ |
| BPP at Point p | 0.852 ^a^ |
| BPP at Point p1 | 0.864 ^a^ |
| BPP at Point p2 | 0.906 ^a^ |
| BPP at Point p3 | 0.874 ^a^ |
| Alveolar bone inclination angle | 0.901^b^ |
| Implant-to-bone angulation | 0.909 ^b^ |

Abbreviation: BPP, buccal plate perforation.

^a^ Coen’s kappa value; ^b^ Intraclass correlation coefficient.

**Supplementary Table 2.** Collinearity Diagnostics for ABIA and IBA

| Variable | VIF | Tolerance | Condition index | Correlation with other variables |
| --- | --- | --- | --- | --- |
| ABIA | 2.186 | 0.458 | 5.63 | 0.85(with IBA) |
| IBA | 2.186 | 0.458 | 5.63 | 0.85(with AIBA) |

Abbreviations: VIF, Variance inflation factor; ABIA, Alveolar bone inclination angle; IBA, Implant-to-bone angle.


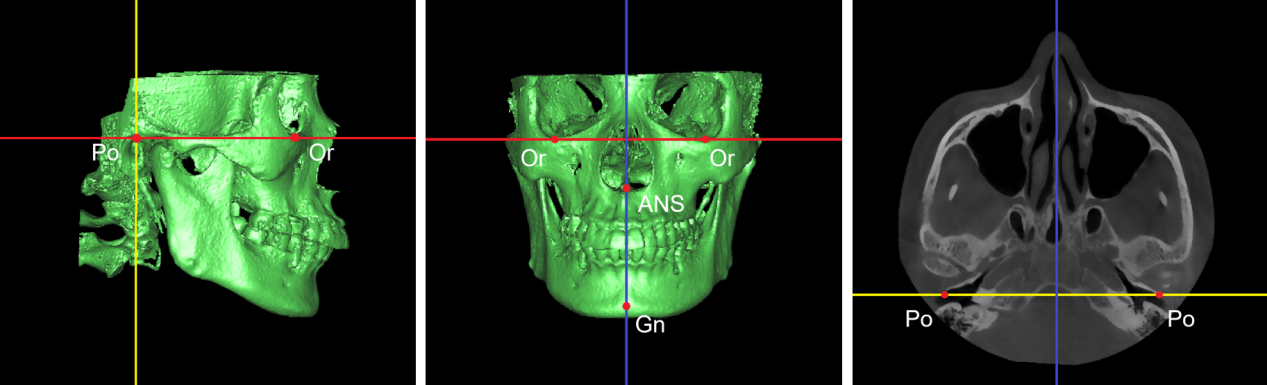


**Supplementary Fig. 1** Determination of reference planes for head reorientation. The axial plane was defined as passing through the left and right orbitale (Or) and the right porion (Po). The coronal plane was defined as passing through both porions and perpendicular to the axial plane. The sagittal plane was defined as passing through the anterior nasal spine (ANS) and gnathion (Gn), and perpendicular to the axial plane.
